# Incidence of gestational trophoblastic disease in South Korea: a longitudinal, population-based study

**DOI:** 10.7717/peerj.6490

**Published:** 2019-02-20

**Authors:** Jin-Sung Yuk, Jong Chul Baek, Ji Eun Park, Hyen Chul Jo, Ji Kwon Park, In Ae Cho

**Affiliations:** 1 Department of Obstetrics and Gynecology, College of Medicine, Eulji University, Nowon Eulji Medical Center, Seoul, Republic of Korea; 2 Department of Obstetrics and Gynecology, College of Medicine, Gyeongsang National University, Gyeongsang National University Changwon Hospital, Changwon, Republic of Korea; 3 Department of Obstetrics and Gynecology, College of Medicine, Gyeongsang National University Hospital, Jinju, Republic of Korea

**Keywords:** Incidence, Gestational trophoblastic disease, Pregnancy

## Abstract

**Introduction:**

We investigated the rate and longitudinal trends of gestational trophoblastic disease (GTD) incidence in the Republic of Korea between 2009 and 2015 using population-based data.

**Materials and Methods:**

Data of patients diagnosed with GTD from 2009 to 2015 were obtained from the Health Insurance Review and Assessment Service/National Inpatient Sample (HIRA-NIS) in the Republic of Korea. The HIRA annually provides the HIRA-NIS, a collection of clinical data from over one million people. For each year, the HIRA-NIS extracted records of 13% of patients admitted at any one time during the year and 1% of all remaining patients using the weighted sample method.

**Results:**

Medical records of 370,117 women with at least one pregnancy (GTD, ectopic pregnancy, abortion, or delivery) were extracted from a total of 4,476,495 records. Of these, 372 episodes of GTD were identified in women with a mean age of 35.4 ± 0.7 years. The incidence rate of GTD was 130 ± 10 cases per 100,000 pregnancies, which was classified as hydatidiform mole (HM), invasive mole, or malignant neoplasm of the placenta with incidence rates of 110 ± 10, 20 ± 0, or 10 ± 0 cases per 100,000 pregnancies, respectively. Incidence of GTD was lowest among women in their late 20 s and early 30 s. Occurrences of HM accounted for 80.3% all GTD cases. Weighted logistic analysis indicated that while age significantly affected the incidence of GTD (odds ratio (OR): 2.46; 95% confidence interval (CI) [1.79–3.37]; *P* < 0.001), socioeconomic status did not (OR: 1.94; 95% CI [1.0–3.79]; *P* = 0.05).

**Conclusions:**

In the Republic of Korea, we observed overall incidence rates of GTD and HM of 1.3 and 1.1 per 1,000 pregnancies, respectively, which are similar to those reported in recent Western population-based studies. We also noted that annual incidence rates of GTD stabilized from 2009 to 2015.

## Introduction

Gestational trophoblastic disease (GTD) is a group of rare gynecologic tumors comprised of heterogeneous types of abnormal placental development. The disease includes complete and partial hydatidiform moles (HM) and gestational trophoblastic neoplasia (GTN), which encompasses choriocarcinomas (CC), placental site trophoblastic tumors, epithelioid trophoblastic tumors, and invasive moles (Lurain, 2010; Seckl, Sebire & Berkowitz, 2010). The exact incidence of GTD is difficult to ascertain because of its rarity and geography-associated variation (Bracken, 1987), as well as inconsistent definitions and diagnostic criteria, inadequate histological examinations, and differences in how births are classified (Eysbouts et al., 2016; Palmer, 1994). Incidence of GTD has decreased over time in Asia (Kim et al., 2004; Matsui et al., 2007), whereas some European countries have reported steady or increased rates (Eysbouts et al., 2016; Joneborg et al., 2018; Lybol et al., 2011). Thus, more recent and accurate data are necessary to evaluate actual incidence rates of GTD.

Our present study investigates the longitudinal incidence rates of GTD in South Korea between 2009 and 2015 using population-based demographic and clinical data. We also compare incidence rates and trends with those in Western population-based studies and reports from South Korea before 2005.

## Materials and Methods

### Study data participants

Most residents of the Republic of Korea (more than 98%) are covered by the National Health Insurance. The Health Insurance Review and Assessment Service (HIRA) reviews nearly all medical claims between the insurance service and medical institutions. The HIRA annually provides the HIRA-National Inpatient Sample (HIRA-NIS), a collection of clinical data from over one million people. HIRA-NIS data from 2009 to 2015 were used for this study (serial numbers 2009-0066, 2010-0084, 2011-0063, 2012-0058, 2013-0085, 2014-0068, and 2015-0057). For each year, we extracted records of 13% of patients admitted at any one time during the year and 1% of all remaining patients using the weighted sample method (Kim, Kim & Kim, 2014).

### Diagnostic and demographic data collection

The Korean Standard Classification of Diseases (7th edition), adapted from the International Statistical Classification of Diseases and Related Health Problems (10th edition) and the HIRA Drug Ingredients Codes and 2016 Health Insurance Medical Care Expenses were used to diagnose GTD. Diagnosis codes for HM (O01.x), invasive HM (D39.2), or malignant neoplasm of placenta (C58.x) denoted GTD, and associated operative procedure codes included those for aspiration biopsies (C8573), simple curettage (C8574), uterine suction curettage (R4481), total hysterectomy (R4482), dilatation, and curettage (R4521), simple hysterectomy with lymphadenectomy (R4143), complex hysterectomy with lymphadenectomy (R4144), simple abdominal hysterectomy (R4145), complex abdominal hysterectomy (R4146), radical hysterectomy with bilateral pelvic lymphadenectomy with para-aortic lymph node biopsy (R4154), radical hysterectomy with bilateral pelvic lymphadenectomy without para-aortic lymph node biopsy (R4155), subtotal hysterectomy (R4130), vaginal hysterectomy (R4202), and vaginal hysterectomy with anterior and posterior repairs (R4203).

Types of pregnancy and/or delivery included ectopic pregnancy (O00.x), other abnormal products of conception (O02.x), spontaneous abortion (O03.x), medical abortion (O04.x), other abortion (O05.x), unspecified abortion (O06.x), normal delivery (O80.x), single delivery by forceps and vacuum extractor (O81.x), single delivery by Cesarean section (O82.x), other assisted single delivery (O83.x), and multiple delivery (O84.x). Antenatal care of normal pregnancy was defined as incidental pregnant state (Z33.x) or supervision of normal pregnancy (Z34.x).

Diagnosis of GTD, ectopic pregnancy, abortion, and antenatal care of normal pregnancy is often not confirmed, so if multiple consecutive diagnostic codes were assigned within 60 days of each other, the last chronological code was used as the final diagnosis. If diagnostic codes were used in intervals greater than 60 days, each code was classified as a distinct diagnosis.

Total number of pregnancies was defined as the sum of GTD cases, ectopic pregnancies, abortions, and deliveries. The incidence rate of GTD was defined as the sum of GTD cases divided by total pregnancies, excluding antenatal care of pregnancy due to overlap with number of deliveries. Low socioeconomic status (SES) was defined as use of a non-general insurance code, such those used for recipients of livelihood programs and homeless individuals.

### Statistical analysis

All statistical analyses were performed with R version 3.3.2 (R Core Team, 2016). All tests were two-tailed and defined as statistically significant when the *P*-value was less than 0.05. Weighted analysis was performed to calculate the incidences and mean ages of the population. The Student’s *t*-test was used to compare continuous variable means, and Pearson’s chi-square or Fisher’s exact test was used to compare categorical variables. Odds ratios (OR) of independent variables were determined with logistic regression analysis.

### Ethical statement

Because this study used data from patients who could not be personally identified, our protocol was not subject to approval by our hospital’s institutional review board according to the Korean Bioethics and Safety Act.

## Results

**Figure 1:**
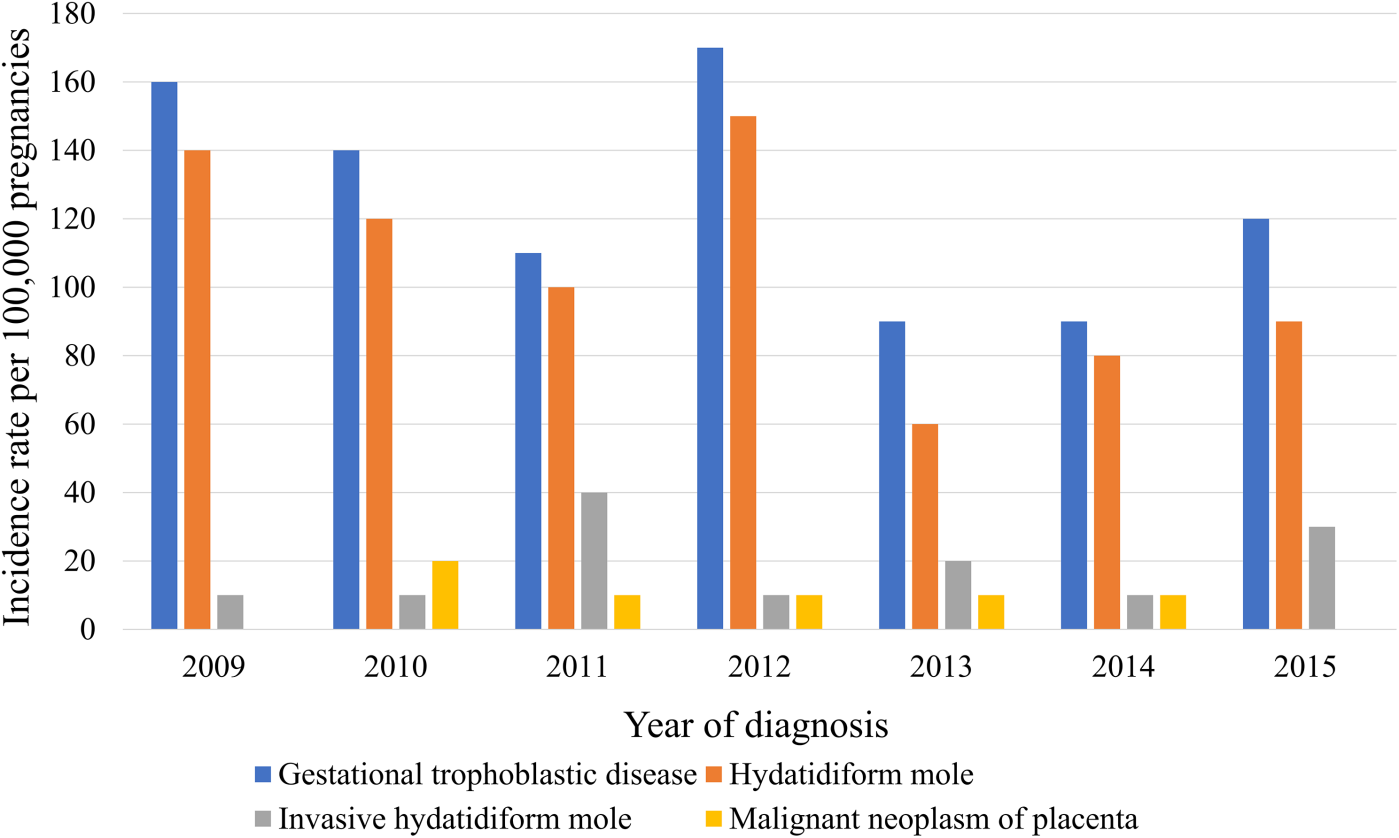
Trending incidence rates of gestational trophoblastic disease, hydatidiform mole, invasive hydatidiform mole, and malignant neoplasm of the placenta in South Korea from 2009 to 2015.

**Figure 2:**
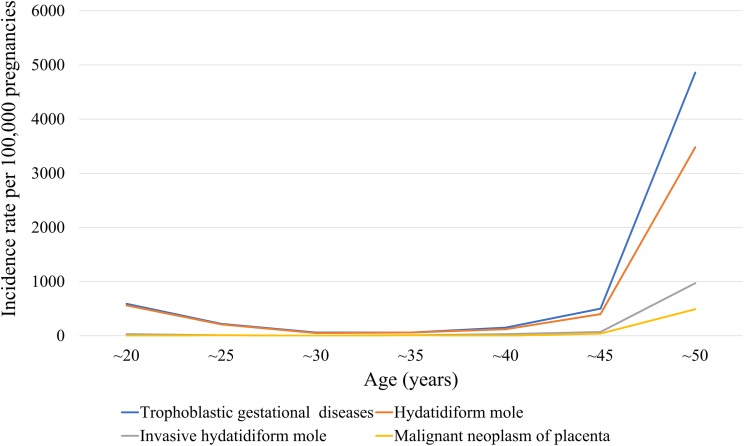
Stratified 5-year patient age ranges of GTD, hydatidiform mole, invasive hydatidiform mole, and malignant neoplasm of the placenta per 100,000 pregnancies in South Korea.

The medical records of 370,117 women with at least one pregnancy were extracted from a total of 4,476,495 patients from 2009 to 2015. We identified 372 episodes of GTD among women with an average age of 35.4 ± 0.7 years (31.1 ± 0 years for those without GTD). Table 1 presents GTD case frequency and type according to year. The incidence rate of GTD between 2009 and 2015 was 130 ± 10 cases per 100,000 pregnancies, which included HM, invasive HM, and malignant neoplasm of the placenta (110 ± 10, 20 ± 0, and 10 ± 0 cases per 100,000 pregnancies, respectively (Table 2)). Figure 1 shows the incidence rates of GTD according to study year, while Fig. 2 illustrates GTD incidence with respect to patient age. The lowest incidence of GTD occurred in patients in their late 20 s and early 30 s and the highest in patients in their late 40 s and beyond. HM accounted for 80.3% of all GTD cases, followed by invasive HM (13.1%), and malignant neoplasm of the placenta (6.6%). According to weighted logistic analysis, age significantly correlated with incidence of GTD (OR: 2.46; 95% CI [1.79–3.37]; *P* < 0.001), but sample year (OR: 0.94; 95% CI [0.8–1.02]; *P* = 0.108) and SES did not (OR: 1.94; 95% CI [1.0–3.79]; *P* = 0.051) (Table 3).

**Table 1:** Annual gestational trophoblastic disease cases in South Korea from 2009 to 2015.

	2009	2010	2011	2012	2013	2014	2015	*P*
	(N* = 62)	(*N* = 68)	(*N* = 53)	(*N* = 61)	(*N* = 55)	(*N* = 40)	(*N* = 33)	
Age	32.6 ± 8.9	37.0 ± 9.1	35.6 ± 9.1	34.9 ± 9.8	38.0 ± 9.5	35.1 ± 10.7	34.9 ± 8.3	0.292
Hydatidiform mole^+^	51 (82.3%)	53 (77.9%)	38 (71.7%)	48 (78.7%)	37 (67.3%)	33 (82.5%)	25 (75.8%)	0.482
Complete hydatidiform mole	4 (6.5%)	6 (8.8%)	5 (9.4%)	11 (18.0%)	6 (10.9%)	4 (10.0%)	2 (6.1%)	0.526^a^
Partial hydatidiform mole	7 (11.3%)	8 (11.8%)	10 (18.9%)	5 (8.2%)	3 (5.5%)	3 (7.5%)	1 (3.0%)	0.255^a^
Invasive hydatidiform mole	9 (14.5%)	5 (7.4%)	11 (20.8%)	5 (8.2%)	10 (18.2%)	4 (10.0%)	6 (18.2%)	0.211^a^
Malignant neoplasm of the placenta	2 (3.2%)	10 (14.7%)	4 (7.5%)	8 (13.1%)	8 (14.5%)	3 (7.5%)	2 (6.1%)	0.226^a^

**Notes:**

* *N* represents the number of cases.

+ Hydatidiform mole includes complete, partial, and unspecified hydatidiform mole.

a Fisher’s exact test was used for data analysis.

**Table 2:** Estimated gestational trophoblastic disease rates per 100,000 total pregnancies from 2009 to 2015 in the Republic of Korea.

	Value ± standard error
Gestational trophoblastic diseases*	130 ± 10
Hydatidiform mole^+^	110 ± 10
Complete hydatidiform mole	10 ± 0
Partial hydatidiform mole	10 ± 0
Invasive hydatidiform mole	20 ± 0
Malignant neoplasm of the placenta	10 ± 0

**Notes:**

* When a patient had more than two gestational trophoblastic conditions, that case was calculated as one episode.

+ Hydatidiform mole includes complete, partial, and unspecified hydatidiform mole.

**Table 3:** Multivariate logistic regression analysis of risk factors for gestational trophoblastic disease.

	Gestational trophoblastic diseases		Hydatidiform mole		Invasive hydatidiform mole		Malignant neoplasm of placenta	
	OR	*P*-value	OR	*P*-value	OR	*P*-value	OR	*P*-value
5-year patient age range	2.46 (1.79–3.37)	<0.001	2.14 (1.45–3.16)	<0.001	3.51 (2.33–5.3)	<0.001	3.22 (2.13–4.88)	<0.001
Sample year	0.9 (0.8–1.02)	0.108	0.87 (0.76–1.01)	0.063	1.04 (0.77–1.4)	0.8	0.93 (0.8–1.08)	0.322
SES	1.94 (1–3.79)	0.051	1.75 (0.81–3.8)	0.156	2.45 (0.66–9.15)	0.182	1.71 (0.22–13.3)	0.608

**Note:**

OR, odds ratio.

SES, socioeconomic status.

## Discussion

Gestational trophoblastic disease consists of various malignancies derived from abnormal trophoblastic proliferation, including complete and partial HM, exaggerated placental site, placental site nodule, invasive HM, CC, placental site trophoblastic tumor, and epithelioid trophoblastic tumor, the latter three of which are considered GTNs (Seckl, Sebire & Berkowitz, 2010). Histologic examination of uterine evacuation specimens is essential for HM diagnosis. Distinguishing among complete mole, partial mole, and hydropic abortus at earlier gestational ages is difficult and sometimes impossible using light microscopy alone. When pathologic findings are also not definitive, accurate diagnosis is performed through ancillary methods, including immunostaining and DNA genotyping (Ronnett, 2018). In our study, diagnosis of HM was made histologically, with unspecified HM in 73.7% of cases, partial HM in 13.3% of cases, and complete HM in 13% of cases. A total of 73.7% of HM cases were coded as unspecified HM with no subcategory code as complete or partial HM. In South Korea, pathologists often do not consider differential diagnostic reports to identify partial and complete HM because this reporting is not mandatory. Unlike other malignant tumors, the diagnosis of GTN is made primarily based on combined clinical presentation and pathologic criteria. Based on GTN subtype, histological confirmation may be problematic in the absence of definitive histologic specimens and should be correlated with serum β-human chorionic gonadotropin levels and radiological findings. GTN is unique in that treatment and surveillance must sometimes be based more on clinical, rather than histological, characteristics of the disease (Lurain, 2010). A centralized pathology review and registration system should be established to ensure more accurate pathologic data and concise diagnostic coding.

The reported incidence of HM varies considerably across different geographic regions (Altieri et al., 2003; Palmer, 1994) due to imprecise definitions and diagnoses of GTD, inadequacy of histological examinations, and inconsistent use of denominators used (e.g., the number of deliveries, live births, pregnancies, etc.) (Eysbouts et al., 2016; Palmer, 1994). Population-based data, rather than earlier hospital-based data, better reflect trends in GTD incidence rates. Determining the total number of pregnancies is difficult, so the number of deliveries or live births are typically used as surrogate parameters, although the total number of pregnancies is the most ideal denominator (Altieri et al., 2003; Joneborg et al., 2018) and should include all live births, stillbirths, abortions, ectopic pregnancies, and clinically unrecognized pregnancies. Use of these specific pregnancy statuses contributes to more accurate estimates of GTD incidence rates across geographic regions (Eysbouts et al., 2016). In South Korea, pregnancy-related diseases are covered by National Health Insurance, so pregnancy is confirmed early, and clinically unrecognized pregnancies are rare. Medical institutions are required to record diagnostic codes and issue a medical certificate for pregnancy-related expenses and social benefits for these women. Therefore, HIRA-NIS data include all diagnostic and procedure codes that may occur during pregnancy, which can help determine the exact incidence rate of GTD in South Korea.

In recent population-based studies, the incidence rate of HM was 1.19 case per 1,000 deliveries in the United States, 1.2 case per 1,000 deliveries in Sweden, and 1.67 case per 1,000 deliveries in the Netherlands (Eysbouts et al., 2016; Lybol et al., 2011; Salehi et al., 2011; Smith et al., 2003), whereas studies in Asian countries reported a wide range of HM incidence rates. For example, incidence of HM in South Korea ranged from 1.9 to 2.1 cases per 1,000 deliveries (Kim et al., 2004, 2007; Martin & Kim, 1998), and incidence of HM in China ranged from 0.81 to 2.5 cases per 1,000 pregnancies (Shang, 1982; Shi et al., 2005). The incidence of HM in Japan ranged from 1.5 to 3.05 cases per 1,000 pregnancies (Matsui et al., 2007; Tangtrakul et al., 1984) Indonesia has one of the highest GTD incidence rates globally with over 10 cases per 1,000 pregnancies (Aziz et al., 1984). Studies in East Asia have suggested incidence rates of 1–3 per 1,000 pregnancies (Atrash, Hogue & Grimes, 1986), although recent data indicate rates lower than or similar those in North America and Europe (Altieri et al., 2003; Brinton, Bracken & Connelly, 1986; Martin & Kim, 1998; Smith et al., 2003; Steigrad, 2003). Longitudinal incidence rates of GTD show a decreasing trend in Asia (Matsui et al., 2007; Shi et al., 2005), while some European countries have experienced increasing rates (Joneborg et al., 2018; Lybol et al., 2011). However, direct comparisons of GTD incidence are difficult to make due to diverse populations and different denominators used in these reports. Age-standardized incidence rates of GTD obtained from cancer registry databases are a valuable means of comparing incidence rates in different areas of the world (Altieri et al., 2003; Smith et al., 2003), so further investigations using such age-standardized data could enhance comparative studies of GTD occurrence.

Incidence rates of HM in South Korea have gradually decreased in reported stratified 5-year increments (cases per 1,000 births): 40.3 in 1971–1975; 16.7 in 1976–1980; 5.4 in 1981–1985; 3.5 in 1986–1990; 2.3 in 1991–1995, 2.1 in 1996–2000, and 1.9 in 2001–2005 (Kim et al., 1998, 2004, 2007; Martin & Kim, 1998). In the present study, we reported an incidence rate of 1.1 cases per 1,000 pregnancies in 2009–2015. Economic development, socio-medical advances, and the national medical insurance program are acknowledged as likely contributors to decreased GTD incidence in South Korea (Kim et al., 2007; Seckl, Sebire & Berkowitz, 2010). GTD incidence rate is reported in 160 cases in 2009, 140 cases in 2010, 110 cases in 2011, 170 cases in 2012, 90 cases in 2013, 90 cases in 2014, and 120 cases per 100,000 pregnancies in 2015. The highest GTD incidence was noted in 2012. However, there was no statistical significant difference in the time trend increase. The annual incidence rates of GTD appear to be stable from 2009 to 2015. In South Korea, a relatively single ethnic nation, the birth rate of women from Southeast Asia due to immigration has been 5% since the early 21st century; the influence of this demographic change on GTD occurrence should be analyzed in future studies.

Maternal age, reproductive and obstetric history, genetic factors, familial clustering, parental blood groups, viral infection, ethnic differences, and environmental and lifestyle factors have been considered as potential etiologic risk factors for development of HM (Gockley et al., 2016a; Parazzini et al., 1991). Extremes of maternal age and a patient’s medical history of GTD are established risk factors for GTD (Altieri et al., 2003). Advanced or adolescent maternal age has consistently correlated with higher rates of complete HM, which has been observed in many countries, including those in Asia, Europe, and North America (Gockley et al., 2016b). Compared to women aged 20–35 years, the relative risk of HM is 1.1–10.0 for women under 20 years old, 3.0–11.0 for women older than 40 years, and 107.0–841.0 for women older than 45 years (Altieri et al., 2003; Parazzini, La Vecchia & Pampallona, 1986; Sebire et al., 2002). Our-age specific incidence rate graph (Fig. 2) shows a J-shaped curve similar to those reported other prior studies. Prior HM predisposes individuals to another molar pregnancy, although subsequent reproductive outcomes in patients with GTD are similar to those in the general population. However, the risk of repeat molar pregnancy is approximately 1–1.7% (Berkowitz et al., 1998; Vargas et al., 2014).

Some evidence has illustrated correlation of SES with incidence of HM, but information regarding the impact of SES is inconclusive yet inextricably linked to other potential risk factors, including nutritional and environmental parameters (Altieri et al., 2003) In Korea, Taiwan, and Japan, the incidence rate of GTD decreases as the gross domestic product increases (Kim et al., 2004; Wei & Ouyang, 1963), but low patient SES did not significantly correlate to GTD incidence rate in this study. The exact relationships between these factors is poorly understood; thus, investigations of other possible GTD etiological risk factors based on case-control studies in different geographic settings are warranted.

Our study has several limitations. First, the HIRA-NIS data used in our study consisted of 1-year sample data that did not include parity information or individual reproductive history, so risk factors other than patient age and SES were not evaluated. Second, data used in this study were based on diagnostic codes without corroborating pathologic findings, so the accuracy of histology-based diagnoses cannot be completely verified. However, HIRA-NIS data are extremely consistent with respect to other gynecology malignancies (Yuk et al., 2016), as National Health Insurance reimburses medical institution only if the diagnosis is confirmed histologically, ensuring few cases of misdiagnosis. Third, Southeast Asian women with relatively high incidence of GTD have accounted for 5% of births in South Korea since the early 21st century, but we could not analyze the impact of these demographic changes on GTD incidence. Despite these limitations, our study yielded longitudinal population-based incidence rates of GTD, including HM using total pregnancies as a comprehensive denominator to include live births, stillbirths, spontaneous abortions, and ectopic pregnancies.

## Conclusions

We determined the incidence rates of GTD and HM as 1.3 and 1.1 cases per 1,000 pregnancies, respectively. The use of all pregnancy occurrences, not just live births, provide accurate information to calculate GTD incidence. Maternal age, but not SES, significantly correlated to GTD incidence, similar to what has been reported in Western populations. Trends of GTD incidence in South Korea have decreased overall compared with studies before 2005, and the annual incidence rates of GTD appear to have stabilized from 2009 to 2015.

## Supplemental Information

10.7717/peerj.6490/supp-1 Supplemental Information 1 Raw data.

## Additional Information and Declarations

### Data Availability

The following information was supplied regarding data availability: The raw measurements are available in the Supplemental File.
